# Development of a Cytocompatible Scaffold from Pig Immature Testicular Tissue Allowing Human Sertoli Cell Attachment, Proliferation and Functionality

**DOI:** 10.3390/ijms19010227

**Published:** 2018-01-12

**Authors:** Maxime Vermeulen, Federico Del Vento, Francesca de Michele, Jonathan Poels, Christine Wyns

**Affiliations:** 1 Gynecology-Andrology Research Unit, Institut de Recherche Expérimentale et Clinique, Medical School, Université Catholique de Louvain, 1200 Brussels, Belgium; vermeulen.maxime@live.be (M.V.); federico.delvento@gmail.com (F.D.V.); francesca.demichele@uclouvain.be (F.d.M.); 2 Department of Gynecology-Andrology, Cliniques Universitaires Saint-Luc, 1200 Brussels, Belgium; jonathan.poels@uclouvain.be

**Keywords:** decellularization, decellularized tissue, fertility preservation, immature testicular tissue, testicular organoid, regenerative medicine, scaffold, extracellular matrix, tissue engineering

## Abstract

Cryopreservation of immature testicular tissue before chemo/radiotherapy is the only option to preserve fertility of cancer-affected prepubertal boys. To avoid reintroduction of malignant cells, development of a transplantable scaffold by decellularization of pig immature testicular tissue (ITT) able to support decontaminated testicular cells could be an option for fertility restoration in these patients. We, therefore, compared decellularization protocols to produce a cytocompatible scaffold. Fragments of ITT from 15 piglets were decellularized using three protocols: sodium dodecyl sulfate (SDS)-Triton (ST), Triton-SDS-Triton (TST) and trypsin 0.05%/ethylenediaminetetraacetic acid (EDTA) 0.02%-Triton (TET) with varying detergent concentrations. All protocols were able to lower DNA levels. Collagen retention was demonstrated in all groups except ST 1%, and a significant decrease in glycosaminoglycans was observed in the TST 1% and TET 1% groups. When Sertoli cells (SCs) were cultured with decellularized tissue, no signs of cytotoxicity were detected. A higher SC proliferation rate and greater stem cell factor secretion were observed than with SCs cultured without scaffold. ST 0.01% and TET 3% conditions offered the best compromise in terms of DNA elimination and extracellular matrix (ECM) preservation, while ensuring good attachment, proliferation and functionality of human SCs. This study demonstrates the potential of using decellularized pig ITT for human testicular tissue engineering purposes.

## 1. Introduction

Thanks to progress in the field of cancer therapy, more than 80% of children now survive their disease in Europe [1]. Unfortunately, cancer treatments induce cytotoxic effects, including gonadotoxicity. While men have the opportunity to cryopreserve a semen sample before starting fertility-threatening therapies, this is not an option for prepubertal boys since no spermatozoa are produced before puberty. Cryopreservation of immature testicular tissue (ITT) containing spermatogonial stem cells (SSCs) is now an ethically accepted approach proposed to these boys with the aim of restoring their fertility once they reach adulthood [2,3]. For now, three methods are conceivable to restore fertility from stored ITT: autotransplantation, in vitro maturation, or SSC transplantation following selective cell isolation. These different approaches are all supported by results obtained in animals, yielding offspring from in vitro-produced mouse spermatozoa [4] or following testicular tissue grafts even in larger animals including non-human primates [5]. Transplantation of isolated SSCs has also led to generation of offspring in mice, rats, goats, chickens, sheeps and zebrafishes [6,7,8,9,10,11], as well as embryo formation in monkeys [12]. However, grafting one’s own tissue or unselected testicular cell suspensions cannot be considered in patients suffering from hematological cancers due to the risk of reintroducing the disease, and in vitro differentiation of human SSCs to spermatozoa has not yet been achieved. Since the start of clinical application of ITT cryopreservation [2,3,13,14,15], many boys have reached the age at which fertility becomes an increasing concern, so development of techniques allowing them to father children is an ever more pressing matter.

Production of male gametes takes place inside the seminiferous tubules of the testis. These coiled tubes contain seminiferous epithelium consisting of germ cells together with their nursing Sertoli cells (SCs), both relying on a specialized form of extracellular matrix (ECM) called the basal lamina. Along with the basal lamina, fibril-embedded layers of myoid cells constitute the lamina propria, which surrounds the seminiferous epithelium to form an enclosed environment suitable for spermatogenesis [16]. This tubular wall allows separation of seminiferous epithelium from the testicular interstitium, where Leydig cells and blood vessels ensure production and transport of androgens [17]. It has been shown that SCs and myoid cells act together to produce the different components required for ECM and basal lamina formation [18]. Notably, laminin and fibronectin are produced by SCs and myoid cells respectively, while collagen IV and proteoglycans are produced by both cell lines [19,20]. Use of ECM-coated surfaces in vitro was found to promote SC and germ cell differentiation [21]. It is also known that the ECM is involved in spermatogenesis, with laminin and collagens allowing differentiating germ cells to cross from the basal lamina to the lumen of seminiferous tubules through regulation of junctional restructuring events [22]. Modifications in the localization of laminin and collagen IV in pathologies such as Sertoli-cell-only syndrome, cryptorchidism and testis atrophy highlight the importance of the ECM for normal functioning of the testis [23,24]. 

Development of a testicular ECM-derived scaffold able to support testicular cells could offer new insights into essential cell–matrix interactions occurring during spermatogenesis. Indeed, production of organ-specific ECMs by tissue decellularization has aroused increasing interest during recent decades, with numerous potential applications like organ/tissue replacement, drug screening or stem cell differentiation studies [25]. The decellularization process allows cell and debris removal, while at the same time preserving matrix composition, biological activity, three-dimensional organization and integrity [26]. Many agents and protocols have been used for appropriate decellularization, but their efficacy depends on tissue type and exposure time to reagents. Commonly used agents can differ widely in nature; they may be physical (freeze/thawing, hydrostatic pressure, sonication), chemical (alcohols, hypo/hypertonic solutions, ionic/non-ionic detergents) or biological, with enzymatic (trypsin, dispase, nuclease) or non-enzymatic (ethylenediaminetetraacetic acid (EDTA)) reagents [25]. The derived scaffold can then be repopulated with stem and somatic cells to produce a functional artificial organ [27]. In the field of reproductive medicine, it may be used in vitro to achieve spermatogenesis and obtain spermatozoa, or in vivo for grafting purposes to restore fertility. Recently, a decellularization protocol was developed for human adult testicular tissue by treating 1 cm^3^ testicular fragments with sodium dodecyl sulfate (SDS) 1%, reported to be the best concentration in terms of DNA elimination [28]. These authors further showed that scaffolds produced from adult and postpubertal testes allowed spermatogonial proliferation and some specific functioning of niche cells [29]. However, no comparison was made between different decellularization protocols and, as far as we know, decellularization of ITT has not yet been investigated. The aim of our study was to determine the best conditions to obtain a decellularized prepubertal testicular tissue matrix able to support human testicular cells. Porcine testicular tissue was chosen since bioengineered pig tissues have already been approved for clinical application [30]. To reach our objective, we compared a total of eight different decellularization protocols in terms of cell and DNA removal, as well as maintenance of testicular tissue structure and important ECM components. The cytocompatibility of decellularized tissues (DTs) showing the best compromise in terms of DNA removal and ECM preservation with human SCs was evaluated by their attachment to the DTs and their proliferative and functional capacities.

## 2. Results

### 2.1. DNA Content Evaluation

**Figure 1:**
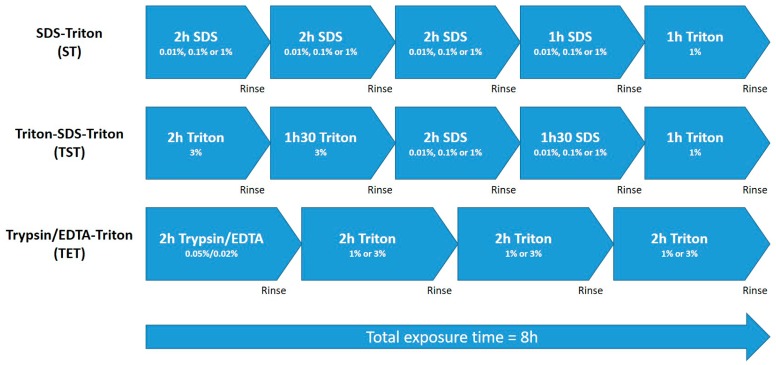
Schematic representation of decellularization processes. Total exposure time to decellularization solutions was eight hours in each case, punctuated by rinse steps in dH_2_O.

**Figure 2:**
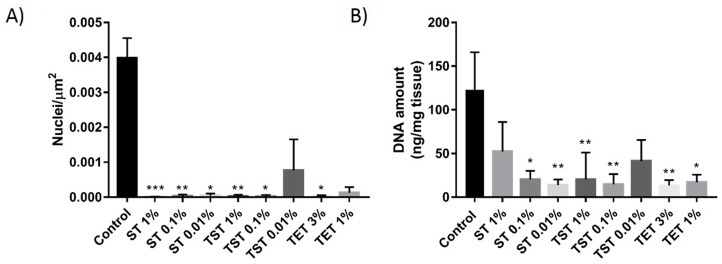
DNA quantification in DT. (**A**) Nuclei were counted on H and E-stained slides of native tissue and DT and represented as the number of nuclei per µm^2^; (**B**) Following tissue digestion, DNA was extracted and quantified using the Quant-iT™ PicoGreen^®^ dsDNA assay. The results are presented as means ± SD. *** *p* < 0.001, ** *p* < 0.01, * *p* < 0.05.

Counting of nuclei on hematoxylin and eosin (H and E)-stained slides from native tissue and the SDS-Triton (ST), Triton-SDS-Triton (TST) and Trypsin/EDTA-Triton (TET) groups (Figure 1) showed a significant decrease in the number of nuclei/µm^2^ in all groups compared to native control tissue, except TST 0.01% and TET 1% (Figure 2A). A significant reduction in DNA levels was achieved in ST 0.01% (13.97 ± 6.34 ng/mg), ST 0.1% (20.21 ± 9.82 ng/mg), TST 0.1% (14.75 ± 11.59 ng/mg), TST 1% (20.23 ± 31.01), TET 1% (17.09 ± 8.73 ng/mg) and TET 3% (13.45 ± 6.05 ng/mg) but not in ST 1% (52.49 ± 33.58 ng/mg) and TST 0.01% (41.41 ± 24.2 ng/mg) relative to native control tissue (123.1 ± 42.88 ng/mg) (Figure 2B).

### 2.2. Composition of the Testicular ECM

**Figure 3:**
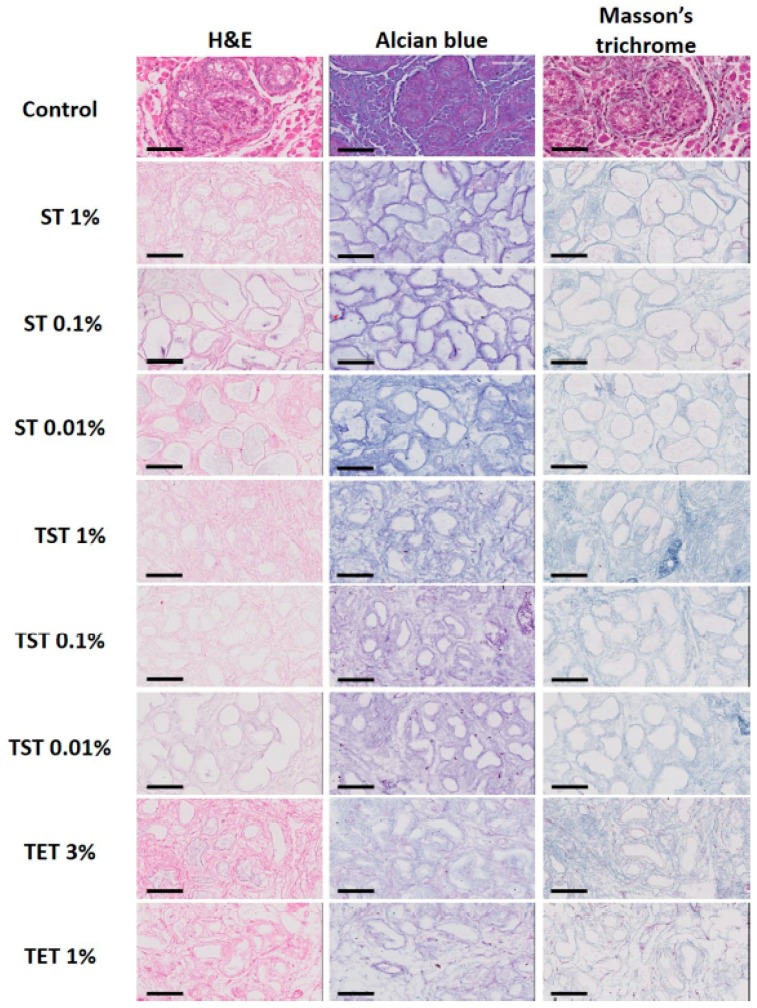
Histological staining of native tissue and DT with different protocols in different conditions. H and E, Alcian blue and Masson’s trichrome staining evidenced preservation of structure, GAGs and collagen respectively. Scale bar = 70 μm.

**Figure 4:**
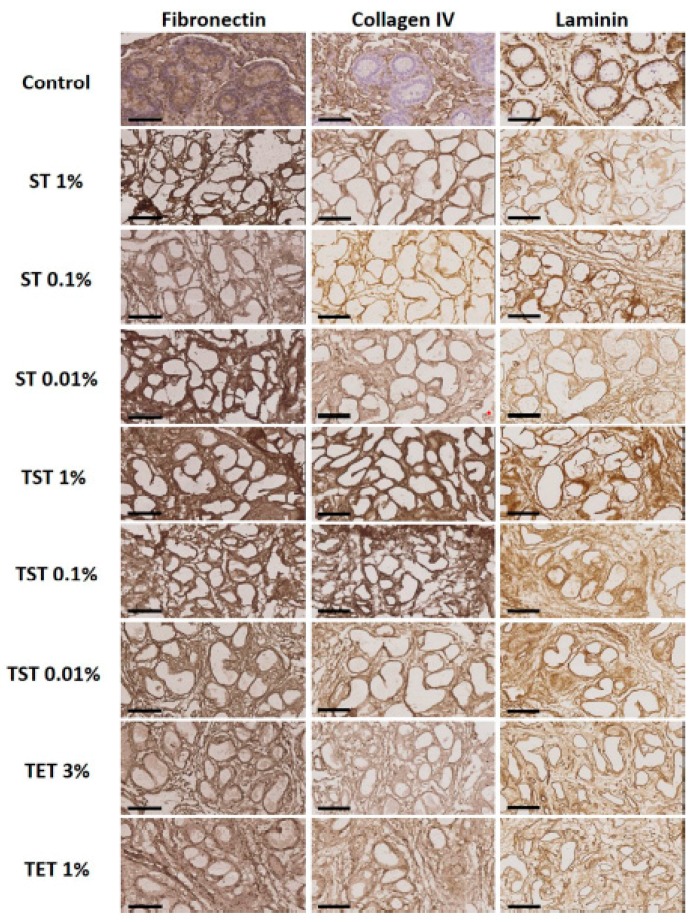
Immunohistochemical analyses of native tissue and DT. Fibronectin, collagen IV and laminin were preserved in tissue decellularized with all protocols in all conditions. Scale bar = 70 µm.

**Figure 5:**
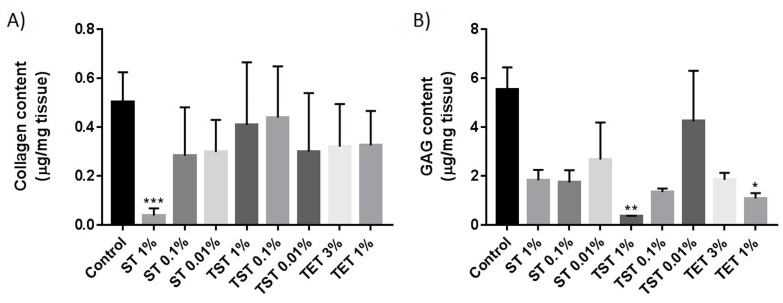
Collagen and GAG quantification in native tissue and DTs. (**A**) Collagen in native tissue and DTs was quantified using the Sircol assay; (**B**) Quantification of GAGs in native tissue and DTs using the Blyscan assay. Results are presented as means ± SD. *** *p* < 0.001, ** *p* < 0.01, * *p* < 0.05.

H and E staining showed a well preserved structural architecture of the testis in all decellularized groups, with empty seminiferous tubules clearly distinguishable (Figure 3). Total collagen and glycosaminoglycans (GAGs) assessed by Masson’s trichrome and Alcian blue staining respectively revealed good retention in all decellularized groups (Figure 3). Figure 4 shows that laminin, fibronectin and collagen IV were clearly detected in native control tissue and in all DTs. Figure 5 illustrates the effect of decellularization on total collagen and GAG levels. A significant reduction in collagen levels was observed only in the ST 1% group compared to controls (Figure 5A). GAG quantification revealed significant loss in the TST 1% and TET 1% groups relative to controls (Figure 5B). 

### 2.3. Scaffold Cytocompatibility

**Figure 6:**
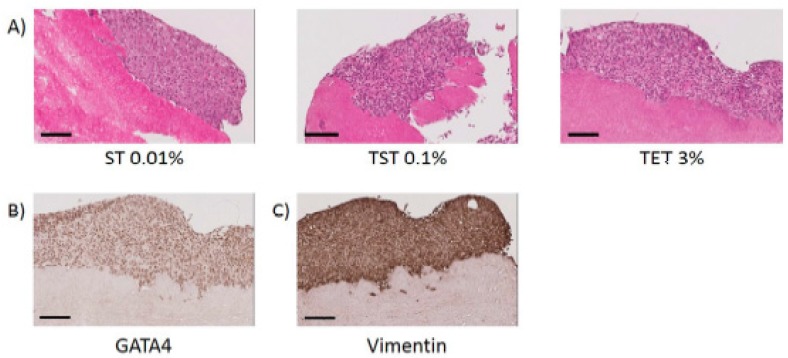
Attachment of SCs onto DTs. (**A**) H and E staining of tissues decellularized with ST 0.01%, TST 0.1% and TET 3% onto which SCs can reattach. Scale bar = 100 µm; (**B**) SCs express GATA4 onto DTs. Scale bar = 100 µm; (**C**) SCs express vimentin onto DTs. Scale bar = 100 µm.

In each of our three groups (ST, TST and TET), we selected ST 0.01%, TST 0.1% and TET 3% for cytocompatibility studies since they allowed best balances between DNA elimination and ECM preservation while using lowest detergent concentrations. Following H and E staining, cell attachment was observable on tissues decellularized with ST 0.01%, TST 0.1% and TET 3% protocols (Figure 6A). Moreover, expression of GATA4 and vimentin by SCs seeded onto DTs was maintained until the end of the culture (Figure 6B,C).

**Figure 7:**
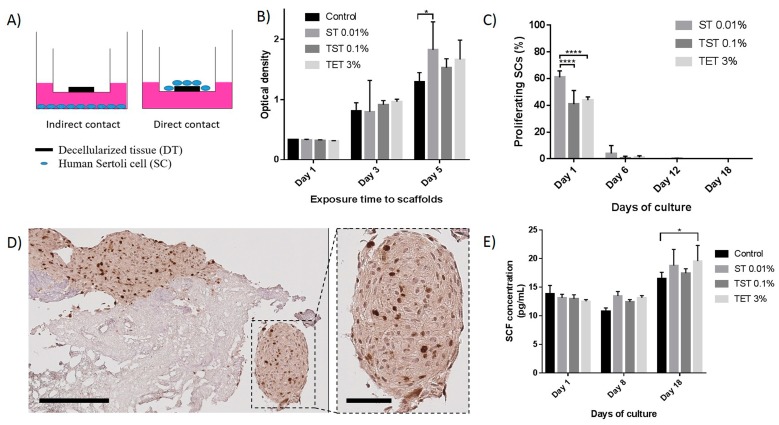
Effects of DTs on proliferation and functionality of SCs. (**A**) Representative drawing of direct and indirect contact culture systems. The indirect contact culture system was used for the WST-1 assay; (**B**) Proliferation of SCs seeded in the compartment below the transwell containing DTs evaluated using the WST-1 assay which is based on the reduction of a tetrazolium salt to a soluble violet formazan product by viable cells. Optical density, which is proportional to cell numbers/well, was recorded on days 1, 3 and 5 using a spectrophotometer; (**C**) Percentage of proliferative SCs seeded onto DTs on days 1, 6, 12 and 18 of culture. SCs seeded onto ST 0.01% DTs present a higher percentage of proliferation compared to TST 0.1% and TET 3% DTs on day 1; (**D**) Immunohistochemical staining of Ki67 in SCs seeded onto DT. The enlarged image shows a round shaped structure as observed in some cases. Scale bares = 200 and 60 µm; (**E**) SCs seeded onto DTs secreted at least the same amount of SCF than SCs seeded alone. Results are presented as mean ± SD. * *p* < 0.05, **** *p* < 0.0001.

Results from the WST-1 assay performed with the indirect contact culture system (Figure 7A) showed that SC proliferation was not negatively affected after one, three or five days compared to control SCs seeded without DTs (Figure 7B). On day 5, cells seeded with ST 0.01% DT showed significantly higher numbers (*p* = 0.0489) (Figure 7B). Cell numbers increased significantly in each group (including control) from day 1 to day 5 (*p* < 0.05). Using the direct contact culture method, quantification of Ki67-positive SCs by immunohistochemistry (IHC) revealed a significantly higher (*p* < 0.0001) percentage of proliferative SCs after one day of culture onto ST 0.01% DTs (60.94 ± 4.85%) compared to TST 0.1% DTs (40.84 ± 10.30%) and TET 3% DTs (43.97 ± 2.29%) (Figure 7C,D). However, these percentages decreased to 4.06 ± 5.81%, 0.73 ± 1.26% and 1.11 ± 1.17% for ST 0.01%, TST 0.1% and TET 3% on day 6 while almost none Ki67-positivity was observed on days 12 and 18. In some cases, SCs formed round shaped structures around the DTs (Figure 7D). 

Stem Cell Factor (SCF) quantified in supernatants recovered during the culture period on days 1 and 8 revealed comparable levels in supernatants originating from SCs cultured on DTs and those seeded alone without DTs. A significantly higher (*p* = 0.0481) SCF concentration was observed on day 18 in TET 3% DT compared to controls (Figure 7E). Lactate dehydrogenase (LDH) was not detected in supernatants after one, eight or 18 days of culture.

## 3. Discussion

ECM scaffolds obtained after tissue decellularization are being increasingly considered for development of artificial organ structures able to mimic organ functions once they have been recolonized by organ-specific cells. Decellularization is defined as a process enabling cell and debris removal, while maintaining the structure and composition of the ECM [26]. However, alteration of ECM due to inappropriate decellularization processes have been demonstrated [31] and combination of different methods and decellularization agents was reported to be a more robust and effective decellularization method [32]. Since the final objective is to create an artificial testis with a decellularized ECM, we were seeking a decellularization process that allows optimal cell and DNA removal, while maintaining scaffold cytocompatbility and preserving the main structure and composition of the testicular ECM. We therefore compared three different protocols (ST, TST and TET), each involving physical (freezing to −80 °C and agitation during the process), chemical (ionic or non-ionic detergents and EDTA) or biological (trypsin) decellularization methods (Figure 1). 

In the absence of any consensus on evaluating decellularization efficiency, assessment of DNA elimination was applied as a first approach. DNA removal was demonstrated by the very small number of nuclei that could be identified on H and E-stained slides, significantly fewer than in native control tissue, except for the TST 0.01% and TET 1% groups (Figure 2A). Because some seminiferous tubules contained diffuse hematoxylin staining without any clear nuclear identification, indicating the potential presence of residual nuclear material, we considered molecular quantification of DNA more relevant. Molecular quantification revealed lower DNA levels, with significant decreases in the ST 0.01%, ST 0.1%, TST 0.1%, TST 1%, TET 1% and TET 3% groups (Figure 2B) ranging from 13.45 ± 6.05 ng/mg (TET 3%) to 20.23 ± 31.01 ng/mg (TST 1%), in line with values obtained for other tissues [33]. To the best of our knowledge, only one other study has investigated decellularization of testicular tissue. Baert et al. used SDS 1% for 24 h to obtain effective decellularization of human adult testicular tissue pieces [28]. DNA removal was also significant, but the exact amount of residual DNA was not communicated.

Preservation of both the structure and composition of the ECM are essential criteria for normal cell behaviour following recellularization. H and E staining showed a well preserved structural architecture of the testis in all decellularized groups, with empty seminiferous tubules clearly distinguishable (Figure 3). Laminin, collagen IV and fibronectin are known to be important proteins in the testicular ECM. Indeed, laminin allows attachment of SCs to the basement membrane [34], laminin and collagen IV are involved in testicular cord formation [35], and fibronectin has been shown to enhance transepithelial electrical resistance of tight junctions between SCs [36]. In this study, IHC evidenced preservation of these proteins after all decellularization protocols (Figure 4). Similarly, Alcian blue and Masson’s trichrome staining demonstrated the presence of GAGs and collagen after decellularization (Figure 3). However, quantitative analyses demonstrated that collagen levels were significantly reduced in the ST 1% group, as were GAG concentrations in TST 1% and TET 1% (Figure 5A,B) relative to controls. Although decreases in collagen and GAGs have already been reported in decellularization protocols using SDS or Triton X-100 without preventing recellularization [37,38], we may assume that seeded cell behavior would be positively impacted if the ECM more closely resembled the intact natural organ ECM in terms of structure and composition. 

In this study, only tissues decellularized with ST 0.01%, TST 0.1% and TET 3% were used for cell attachment, proliferation and functionality analyses, as they represented the best compromise between DNA elimination and ECM preservation. SCs were used for this evaluation because they provide support for initial formation of testicular cords during embryogenesis of the testis, thus playing a key role in the organization and differentiation of other cells [39].

The capacity of SCs to adhere to decellularized seminiferous tubules was previously demonstrated in rats [40]. By means of H and E staining, we showed that human SCs were able to attach to pig ITT decellularized with ST 0.01%, TST 0.1% or TET 3% (Figure 6A) while maintaining expression of GATA4 and vimentin until the end of the culture (Figure 6B,C). Use of testicular tissue originating from pigs is a good alternative, since procuring human ITT for development of a decellularized scaffold is not appropriate for obvious reasons. In this study, wild-type pigs were used for experiments, but use of tissue from alpha 1,3 galactosyltransferase knockout (α-gal KO) animals should be considered in the future, as they were reported to be less immunogenic following xenografting [41]. More importantly, no differences were evidenced between decellularization and recellularization processes in wild-type or α-gal KO tissues [42], suggesting that protocols developed in this study could be applied to tissues originating from α-gal KO animals. Moreover, decellularized ECMs of porcine origin already have various clinical applications in fields like dentistry, orthopedics, plastic/reconstructive surgery and cardiovascular medicine [30].

Analyses of supernatants from SCs cultured on DTs detected no LDH, indicating that DTs are not cytotoxic for SCs. Furthermore, using an indirect contact culture system, we showed that SC numbers were not negatively affected after one, three or five days compared to control SCs seeded without DTs (Figure 7B). The number of cells increased significantly in each group from day 1 to day 5. Compared to control, SC numbers significantly rose only on day 5 when co-cultured with ST 0.01% DT (*p* = 0.0489), proving their proliferation capacity in the presence of the scaffold (Figure 7B). This observation further suggests that DTs still contained soluble molecular factors able to promote SC growth. 

Study of SCs proliferation onto DTs also revealed a significantly higher (*p* < 0.0001) percentage of proliferative SCs onto ST 0.01% DTs compared to TST 0.1% DTs and TET 3% DTs after one day of culture (Figure 7C,D). However, the important decrease of proliferative activity observed on day 6, 12 and 18 could be explained by the contact inhibition mechanism happening when primary cells reach high confluence in vitro or to the basic composition of the culture media. This effect could be circumvented using SCs stimulating factor such as follicle-stimulating hormone which was shown to partially overcome contact inhibition mechanism of SCs cultured at high densities [43]. In the testis, SCF is produced by nursing SCs and is an activator of spermatogonial proliferation [44]. SCF quantified in supernatants recovered during the culture period revealed comparable levels in supernatants originating from SCs cultured on DTs and those seeded alone without DTs indicating that SCs did not lose their functionalities onto DTs (Figure 7E). Interestingly, we observed that human SCs secreted higher amounts of SCF after 18 days of culture on TET 3% DT than cells seeded alone (*p* = 0.0481), suggesting that TET 3% DTs provides structural and/or molecular signals favorable for SCs.

While preparing this manuscript, another study was published reporting culture of human testicular cells on human testicular tissue decellularized with SDS 1% over the course of 24 h [29]. The authors showed that both somatic and spermatogonial cells were able to attach to the scaffold to form an organoid, and that all cell types could be maintained for at least four weeks in vitro. They demonstrated the functionality of cells by hormonal secretion of inhibin B and testosterone. By contrast with our results that showed differences between SCs seeded alone and onto DTs, no difference was observed between cells seeded onto scaffolds or not. This may be due to the fact that ECM components were better preserved in our study, suggesting that decellularization using SDS 1% for 24 h is maybe not the most appropriate approach to improve cell functionality after recellularization. This assumption is further supported by the differences that we observed between SCs behavior when seeded onto different DTs. Another important observation is that in both studies (our and theirs), cells were able to attach to the DTs, but appeared unable to recreate a structure resembling that of normal testicular tissue neither to colonize empty seminiferous tubules. However, round shaped structures formed by SCs aggregation were occasionally observed in our study which could be seminiferous cord-like structures similar to seminiferous cords that are found during testicular development [39]. Such structures were already described by Hadley et al., when SCs where cultured into reconstituted basement membrane [21]. Further studies should, therefore, focus on improving the structural rearrangement of cells seeded onto DTs, possibly by use of a hydrogel developed from decellularized testicular ECM. This method was very recently applied to form organoids using testicular cells from elderly patients [45]. Since immature testicular cells have the capacity to form seminiferous tubules in vitro, as recently demonstrated [46], it may be hypothesized that seeding of prepubertal cells in such organoids could enhance cell colonization and cellular rearrangement properties.

## 4. Materials and Methods

### 4.1. Decellularization of Pig Testicular Tissue

A total of 15 prepubertal pig testes (<21 days) were obtained from the Department of Experimental Surgery and Transplantation (CHEX) of the Catholic University of Louvain (UCL) at the time of animal euthanasia in the context of other studies and following approval by the Committee on Animal Research of the Catholic University of Louvain. The recovered tissue was cut into pieces of ~5 mm^3^, frozen to −80 °C in phosphate-buffered saline (PBS), and stored until use. After thawing in a 37 °C water bath, the tissue fragments were washed for 15 min in 2XPBS and then subjected to either SDS (VWR Chemicals, Leuven, Belgium, 33629.266)-Triton (Sigma-Aldrich, Overijse, Belgium, X-100), Triton-SDS-Triton, or trypsin 0.05%/EDTA 0.02% (Sigma-Aldrich, T4174)-Triton treatment (Figure 1). In each of the three protocols, total exposure time to decellularization solution was 8 h, with each step separated by a rinse cycle involving 5 min in deionized water (dH_2_O) followed by 15 min in 2× PBS. Concentrations of 1%, 0.1% and 0.01% SDS were tested in the ST and TST protocols (abbreviated ST 1%, ST 0.1%, ST 0.01%, TST 1%, TST 0.1% and TST 0.01%), and 3% and 1% Triton in the TET protocol (abbreviated TET 3% and TET 1%). Once the decellularization processes were complete, tissues were rinsed for 15 min in PBS and used for different analyses.

### 4.2. Histology 

Both normal tissue fragments and DTs were fixed in 4% paraformaldehyde (VWR, Leuven, Belgium, 9713 for 24 h at room temperature (RT)). They were alcohol-dehydrated, immersed in xylene and embedded in paraffin. The tissues were then cut into 5 µm-thick sections, followed by deparaffination and rehydration steps by means of toluene and alcohol baths. H and E staining was realized by immersion of slides in different baths in this order: tap water, distilled water, Mayer’s Hematoxylin (Sigma-Aldrich, MHS32), tap water, distilled water and in erythrosin B (VWR, 1.159.360.025). Masson’s trichrome was realized by immersion of slides in different baths in this order: tap water, distilled water, Mayer’s Hematoxylin, tap water, distilled water, ponceau (Sigma-Aldrich, P2395,)-fuchsin (VWR, 1052310025) solution, distilled water, phosphomolybdic acid (Sigma-Aldrich, 79560), distilled water and in methylene blue (Merck, Overijse, Belgium, 1163160050). Following staining procedures, slides were dehydrated and mounted using Dako mounting medium. General observation of slides were realized using a standard optical microscope (E200LED, Nikon, Amsterdam, The Netherlands). The slides were then scanned with the Leica SCN400 slide scanner (Leica Biosystems, Wetzlar, Germany) and images were captured using Aperio Imagescope software (Leica Biosystems, Vista, CA, USA).

### 4.3. Assessment of Nuclear Material Content 

Sections of native tissue and DT were stained with H and E or Masson’s trichrome to evaluate the presence of nuclear residues and general tissue morphology. The efficacy of decellularization was assessed by residual nuclear material quantification on H and E-stained slides. Nuclei were counted in 10 random fields per slide and results were expressed as the number of nuclei per µm^2^. For quantification, DNA was extracted using the PureLink™ genomic DNA mini kit (Thermo Fisher, Ghent, Belgium, K1820-01) and quantified using the Quant-iT™ PicoGreen^®^ dsDNA assay kit (Thermo Fisher, P7589) following the manufacturer’s instructions. 

### 4.4. Immunohistochemistry 

Preservation of fibronectin, collagen IV and laminin were evaluated by immunohistochemistry. Briefly, following deparaffination and rehydration, endogenous peroxidase activity was inhibited by incubation with 0.3% H_2_O_2_ (Perhydrol^®^, Merck, 107209) for 30 min at RT. After washing in dH_2_O, sections were subjected to antigen retrieval steps. For fibronectin and collagen IV antibodies, the slides were placed in citrate buffer for 30 min at 98 °C, while for laminin, they were incubated for 20 min with proteinase K (20 µg/mL, Sigma-Aldrich, Belgium, P6556) at 45 °C. Nonspecific reactions were blocked with 10% normal goat serum (NGS, Thermo Fisher, 31873) and 1% bovine serum albumin (BSA, Sigma-Aldrich, A7030) for 30 min at RT.

Primary antibodies (collagen IV, ab6586, 1/100; fibronectin, ab23750, 1/400; laminin, ab11575, 1/200; and GATA4, ab84593, 1/1600 all from Abcam, Cambridge, UK as well as Ki67, M7240, 1/100 and vimentin, M0725, 1/800; from DAKO, Heverlee, Belgium) were diluted in NGS 1% and BSA 0.1% and incubated over night at 4 °C. The next day, secondary anti-rabbit or anti-mouse antibody (Envision+ system-labeled polymer-horseradish peroxidase (HRP); DAKO, K4003 or K4001) was added for 60 min at RT. Diaminobenzidine (DAKO, K3468) was used as a chromogen. Following brief counterstaining with Mayer’s hematoxylin (DAKO, S3301), the slides were mounted with Dako mounting medium (Dako, C5703). 

### 4.5. Glycosaminoglycan Quantification

Five µm-thick sections of native tissue and DT were deparaffinized (see Section 4.2) and stained with Alcian blue (Bio-Optica, Milan, Italy, 04-160802) to evaluate GAG retention, according to the manufacturer’s instructions. Concentrations of GAGs in native tissue and DT were determined using the Blyscan assay (Tebu-Bio, Boechout, Belgium, B1000) following suppliers’instructions. Briefly, GAGs were extracted from tissues using a sodium-phosphate solution containing papain (Sigma-Aldrich, P3125). Blyscan dye reagent was added to standards (bovine tracheal chondroitin 4-sulfate) as well as to GAGs extracted from native tissue and DTs for 30 min then centrifuged. GAGs-dye complexes were dissociated using the dissociation reagent and the resulting solutions were transferred in 96-well plate followed by measurement of absorbance at 656 nm. GAGs concentrations were determined from the standard curve and results were expressed in µg GAGs/mg tissue.

### 4.6. Collagen Quantification 

Collagen preservation was assessed in native tissue and DT by Masson’s trichrome staining and collagen content using the Sircol assay (Tebu-Bio, S1000) (which allows detection of collagen types I to V) according to suppliers’instructions. The Sircol assay was performed following overnight enzymatic digestion of tissues with 250 U/mL pepsin (Sigma-Aldrich, P6887) in acetic acid (0.5 M, Merck, 100062) to release collagen in the solution. The next day, Isolation and Concentration Reagent was added to sample and incubated for one night at 4 °C. Sircol dye reagent was added to standards (bovine skin collagen) and to collagen extracted from native tissues and DTs for 30 min then centriguged. Samples were then washed with ice-cold Acid-Salt Wash Reagent and centrifuged. The resulting pellets were mixed with Alkali Reagent and vortexed until complete dissolution. Solutions were transferred in 96-well plate for absorbance measurement at 555 nm. Collagen concentrations were determined from the standard curve and results were expressed in µg collagen/mg tissue. 

### 4.7. Cell Culture 

Primary human Sertoli cells were purchased from Lonza (Lonza, Verviers, Belgium, MM-HSE-2305) who informed us that cells were isolated from an adult patient, cultured for three passages and still expressed SOX9 and GATA4 proteins (see certificate of analysis in Supplementary materials). Cells were thawed and cultured in Dubelcco’s modified Eagle’s medium: nutrient mixture F-12 (1:1) supplemented with 15 mM HEPES (4-(2-hydroxyethyl) piperazine-1-ethanesulfonic acid) buffer, 2.5 mM l-glutamine (Lonza, 12-719F) and 5% fetal bovine serum (Sigma-Aldrich, F6178) for one week. The cells were then detached using a 0.02% solution of EDTA (Versene^®^, Lonza, 17-711E) and used for experiments.

### 4.8. SC Proliferation in Indirect Contact with DTs

Approximately 3 × 10^3^ human SCs were seeded per well of 24-well plates, while scaffolds were placed in a Transwell permeable support (Sigma-Aldrich, CLS3413) just above the cells. Cell proliferation was evaluated with cell proliferation reagent WST-1 (Sigma-Aldrich, 000000005015944001), which is based on reduction of tetrazolium salt to soluble formazan. After 1, 3 and 5 days of culture, the WST-1 reagent was added to each well, followed by two hours of incubation at 37 °C with 5% CO_2_. After gentle pipetting, the solutions were transferred to a 96-well plate and their optical density was measured at 450 nm using a spectrophotometer. 

### 4.9. Scaffold Preparation and Culture

Sterilization of DTs scaffolds was realized by agitation in 0.1% peracetic acid (Sigma-Aldrich, 77240)/4% ethanol (VWR, 20821.310) at 4 °C, rinsed three times in PBS (Lonza, BE17-516F) and incubated in culture medium for one night. A 25 μL drop containing 7.5 × 10^4^ SCs was seeded onto each DT scaffold placed in Transwell supports (Millicell^®^ cell culture inserts, Merck, PICM01250) in 24-well plates (BD, Erembodegem, Belgium, 353047). The seeded DT scaffolds were cultured at 37 °C with 5% CO_2_ for a maximum of 18 days. 

### 4.10. SC Attachment, Proliferation, Functionality and Viability on Scaffolds

To evaluate their attachment to and functionality on DT, human SCs were cultured at 37 °C with 5% CO_2_ on each DT fragment for 18 days. The DTs were then recovered and fixed in 4% paraformaldehyde on days 1, 6, 12 and 18, and cell attachment was examined on H and E stained sections. Percentages of proliferative SCs onto DTs were determined using the ratio (number of Ki67 positive cells/total number of cells) × 100. To assess cell functionality at the beginning, in the middle and at the end of the culture period, supernatants recovered on day 1, 8 and 18 were used for stem cell factor SCF quantification by enzyme-linked immunosorbent assay (ELISA) (Bio-Techne, Abingdon, UK, DCK00). Scaffold-induced cytotoxicity was evaluated by quantification of LDH in supernatants on days 1, 8 and 18 from DT cultures using the Pierce™ LDH cytotoxicity assay kit (Thermo Fisher, 88953). 

### 4.11. Statistical Analyses 

Statistical analyses were performed using GraphPad Prism 7 (GraphPad software, La Jolla, CA, USA). Data are presented as means ± SD. Nucleus counting (*n* = 6), DNA (*n* = 6), collagen (*n* = 6) and GAG quantification (*n* = 3) results were analyzed against their respective controls using the Kruskal-Wallis test with Dunn’s post hoc correction. For WST-1 assay (*n* = 3), percentage of proliferative SCs (*n* = 3) and SCF secretion (*n* = 3) analyses, two-way analysis of variance was used, followed by Tukey’s post hoc test for correction of multiple comparisons. Each experiment was performed once using material from n animals.

## 5. Conclusions

In conclusion, we investigated decellularization of pig ITT by comparing three protocols (ST, TST and TET), each involving different detergent concentrations. Evaluation of decellularization efficiency by DNA quantification and preservation of ECM components allowed us to determine ST 0.01%, TST 0.1% and TET 3% as the condition to use out of each protocol, which were then subjected to cytocompatibility studies. Although the three selected protocols resulted in SC attachment, DTs obtained with ST 0.01% allowed a higher SCs proliferation and demonstrated sustained SCs functionality. Use of a solubilized form of DT scaffolds obtained with ST 0.01% could further improve cellular organization and would be a valuable tool for the study of seminiferous tubule formation and development. 

## Supplementary Materials

Supplementary materials can be found at www.mdpi.com/1422-0067/19/1/227/s1.

## Abbreviations

α-gal KO	alpha 1,3 galactosyltransferase knockout
BSA	bovine serum albumin
DNA	deoxyribonucleic acid
DT	decellularized tissue
ECM	extracellular matrix
EDTA	ethylenediaminetetraacetic acid
ELISA	enzyme-linked immunosorbent assay
GAG	glycosaminoglycan
H and E	hematoxylin and eosin
HEPES	4-(2-hydroxyethyl) piperazine-1-ethanesulfonic acid
HRP	horseradish peroxidase
IHC	immunohistochemistry
ITT	immature testicular tissue
LDH	lactate dehydrogenase
NGS	normal goat serum
PBS	phosphate buffer saline
SC	sertoli cell
SCF	stem cell factor
SDS	sodium dodecyl sulfate
SSC	spermatogonial stem cell
ST	SDS-Triton
TET	Trypsin/EDTA-Triton
TST	Triton-SDS-Triton
