# Supplementary material for: Development of a Cytocompatible Scaffold from Pig Immature Testicular Tissue Allowing Human Sertoli Cell Attachment, Proliferation and Functionality
Source: Int J Mol Sci. 2018 Jan 12;19(1):227. doi: 10.3390/ijms19010227 (PMC5796176; doi:10.3390/ijms19010227)
Supplement: Supplementary file 1 [file ijms-19-00227-s001.pdf]

Lonza Walkersville Inc.
8830 Biggs Ford Road
Walkersville, MD 21793 8415
Tel (301) 898 7025
Fax (301) 845 4024

Lonza

Printed on, 29-Sep-2016 08:48

CERTIFICATE OF ANALYSIS

**Product Code:**	MM-HSE-2305	**Lot Number:**	230501081
**Product:**	Human Sertoli Cells	**Manufacture Date:**	02-Jan-2008

**TEST (Method)**	**SPECIFICATIONS** Min.	Max.	Results
Donor Screen Information:			
Age	***	***	23 Y
Sex	***	***	MALE
Race	***	***	C
Cell Type	***	***	PRIMARY SERTOLI
Date of Cryopreservation	***	***	02 JAN 2008
Cell Passage Frozen			3
Viability-Tryp.Blue Exclusion	>= 70%	***	91,00 %
Cell Count (cells/amp)	>= 500,000	***	538000
Total Population Doublings	>= 8	***	11
Seeding Efficiency	>=50%	***	83 %
Doubling Time (days)	2.0	5.0	3,5 Days
SOX9 (FACS)	70	100	97 %
GATA4 (FACS)	70	100	98 %
Sterility	Negative	Negative	Negative
Direct Plating (Mycoplasma)	***	***	Negative
Hoechst Stain Procedure	***	***	Negative
V[illegible]us Testing:			
[illegible]V Test	***	***	Not detected
HBV Test	***	***	Not detected
HCV Test	***	***	Not detected

Details concerning the use of our cell and media products can be downloaded from our website at www.lonza.com/cell-protocols.

This lot has been reviewed by Quality Assurance in compliance with requirements of Lonza's Quality System.
This document was generated from a validated Part 11-compliant electronic system and thus handwritten signatures are not required.
